# The Role of Movement on the Development of the Audiotactile Temporal Binding Window

**DOI:** 10.1111/desc.70191

**Published:** 2026-04-13

**Authors:** Monica Gori, Maria Bianca Amadeo, Margherita Sturlese, Martina Riberto, Anna Vitale, Jessica Bertolasi, David A. Tovar, David J. Lewkowicz, Micah M. Murray, Mark T. Wallace, Helene Vitali

**Affiliations:** ^1^ Unit for Visually Impaired People Istituto Italiano di Tecnologia Genoa Italy; ^2^ DIBRIS University of Genova Genoa Italy; ^3^ Multisensory Research Lab, Department of Psychology and Vanderbilt Brain Institute Vanderbilt University Nashville USA; ^4^ Child Study Center Yale School of Medicine New Haven Connecticut USA; ^5^ The Lausanne University Hospital and University of Lausanne, Lausanne, Tennessee, Switzerland; ^6^ The Sense Innovation and Research Center Lausanne and Sion Switzerland

**Keywords:** active and passive movement, development, school‐age children, time perception, TOJ

## Abstract

The perception of temporal correspondence between sensory signals from body movements and those arising through environmental interactions is crucial for multisensory integration and adaptive behavior. In adults, the perception of multisensory temporal alignment is more accurate during active than passive or no movement. Currently, it is not known whether this is the case in children. Thus, we used an audiotactile temporal order judgment task (TOJ) to investigate multisensory temporal perception during active and passive arm movement in 6 to11‐year‐old blindfolded children (*N* = 87). Results indicated that temporal precision during active movement improved relative to passive movement only in younger children (6‐year‐olds). The active‐passive difference diminished with age, with precision in the passive condition improving. Conversely, the temporal bias developed similarly in active and passive conditions, showing an age‐related decrease, whereby tactile stimuli must precede auditory ones to be perceived as simultaneous. The study suggests that the maturation of the audiotactile temporal perception during movement involves two distinct mechanisms: an early sensorimotor integration of predicted sensory feedback and a faster‐developing multisensory integration aligned with kinesthetic feedback, each following different developmental trajectories.

**Summary:**

- We proposed a task to study whether active and passive movements affect temporal perception of audiotactile stimuli in children.
- Temporal precision increased during the active than passive movement in younger children (6‐year‐old).
- The active‐passive difference diminished with age, with precision in the passive condition improving.
- The maturation of the audio‐tactile perception during movement involves the sensorimotor and multisensory integration mechanisms with two different developmental trajectories.

## 1 Introduction

In daily life, interactions with the environment continuously expose us to multisensory cues, which are integrated by the brain to enhance perception and behavioral performance. For example, response times are faster when congruent auditory and tactile stimuli are presented simultaneously, compared to when only one is provided (Murray et al. 2005; Tajadura‐Jiménez et al. 2009; Zampini et al. 2007). The spatial and temporal alignment of these stimuli plays an important role in whether they will be integrated into a unified perceptual experience or not (Stein and Stanford 2008). Multisensory interaction is further challenged by body movements, which produce sensory feedback requiring precise alignment with environmental cues. During development, this process is particularly complex, as sensorimotor and multisensory mechanisms are still maturing and may exhibit misalignment. However, the impact of active motion on developmental multisensory temporal binding remains largely unexamined. The present study investigates these developmental dynamics between motor and audiotactile temporal interactions.

The temporal aspects of multisensory processing can be investigated by measuring the size of the Temporal Binding Window (TBW) and the Just Noticeable Difference (JND). TBW is the period of time during which stimuli from different senses are integrated and perceptually bound (Wallace and Stevenson 2014). The JND is the smallest temporal interval between two stimuli that a participant can reliably detect as having occurred in a specific order (Keetels and Vroomen 2012). Both measures reflect temporal sensitivity, with larger JNDs generally corresponding to wider TBW, indicating reduced precision in distinguishing the timing of multisensory events. The TBW exhibits adaptive properties to compensate for the biologically significant differences in neural propagation times between sensory systems (Wallace and Stevenson 2014). The size of the TBW can be modulated by multiple factors such as the type of cue (Eijk et al. 2008; Stevenson and Wallace 2013), the environment (Fujisaki et al. 2004; Keetels and Vroomen 2008; Opoku‐Baah and Wallace 2020; Stetson et al. 2006; Wang et al. 2025), individual differences (Stevenson et al. 2012), and age (Ampollini et al. 2024; Basharat et al. 2019). Specifically, during development, the multisensory TBW is broader in infants and continues to change until adolescence (Ampollini et al. 2024; Lewkowicz 1996; Lewkowicz and Flom 2014; Wallace and Stevenson 2014), with size varying according to the nature of the combined stimuli. For instance, the audio‐tactile TBW in school‐age children (7 to 11 years‐old) ranges from 172 to 271 ms, with 9‐year‐olds exhibiting adult‐like values despite greater variability among younger children (Ampollini et al. 2024; Stanley et al. 2019).

The temporal aspects of developmental multisensory processing differ not only in the size of TBW but also in its asymmetry (Lewkowicz 1996). This asymmetry can be measured by calculating the point of subjective simultaneity (PSS). The PSS refers to the temporal separation required for an individual to report that two stimuli are perceptually synchronized; although ideal observers exhibit a PSS of 0 ms (i.e., objective synchrony), empirical observations indicate there is often a bias for one sense temporally leading the other to result in perceived simultaneity (Ampollini et al. 2024; Love et al. 2013; Stanley et al. 2019). Specifically, for audiotactile pairings, the PSS is already shifted in infancy toward the tactile‐leading side (Ampollini et al. 2024).

Though it is well established that the TBW changes with age, some studies describe its gradual refinement (Chen et al. 2016), while others suggest a sequence of plastic phases followed by periods of stability (Ampollini et al. 2024; Hillock‐Dunn and Wallace 2012). This developmental process with alternating phases of plasticity and stability might be associated with the need to align the development of sensory systems with a body that is growing rapidly at times and more slowly at others, while also adapting to the maturation of the motor system.

In adults, research indicates that movement influences audiotactile temporal processing (Kitagawa et al. 2016; Kwon and Miyake 2023; Nishi et al. 2014; Tanaka et al. 2021). Different temporal judgment patterns are observed during active movement compared to passive movement and the absence of movement, suggesting that voluntary action enhances temporal sensitivity and modulates temporal perception simultaneity between sensory stimuli. However, the results are not always consistent across studies (Frissen et al. 2012; Hao et al. 2015). The mechanism that seems to better explain this movement benefit involves the integration of multisensory signals with motor‐related signals, including both kinesthetic feedback (KF) and predicted sensory feedback (PSF), during active movement (Kitagawa et al. 2016). This internal feedback arises from a copy of the efferent motor commands generated in the presupplementary motor cortex and premotor cortex (Christensen et al. 2007; Haggard 2009; von Holst and Mittelstaedt 1950; Wolpert and Ghahramani 2000). Computationally, PSF results from a feed forward internal model that utilizes motor commands as inputs to predict the sensory consequences of planned movements, subtracting this prediction from incoming afferent signals to derive an exafferent input that reflects the environmental state (Gori et al. 2012; Sciutti et al. 2010; von Holst 1954). As a result, the active movement could produce a gain in the temporal precision between sensory cues.

To date, how and when during development interactions between multisensory integration and active and passive motor‐related signals occur remain incompletely understood. The study of the interplay between motor and perceptual development is critical for guiding the design of effective educational and rehabilitative interventions appropriate to different stages of development, while also enhancing our understanding of sensorimotor disorders in children. Here, we address this scientific question by examining how audiotactile temporal precision and accuracy develop in typically developing sighted children aged 6 to 11 years by using a multisensory TOJ task under both active and passive movement conditions.

## 2 Materials and Methods

### 2.1 Participants

We recruited 136 children, ranging in age from 6 to 11 years, at the elementary school of Bolzaneto in Genoa (Italy). Some participants (*N* = 43) were *a* *priori* excluded because they failed to complete the tasks or because their JND exceeded 1000 ms (1000 ms represents the maximum audiotactile temporal delay within a trial). Thus, 93 children were initially included in the data analysis; however, after the removal of statistical outliers (see Table 1 and Results section for details), the final sample consisted of 87 children. Children whose birthdays fell in the same month as the task administration were assigned to the next age group. Additionally, for statistical reasons (i.e., to ensure comparable sample sizes across age groups), the 10‐year‐old group includes five children who had turned 11 no more than three months before testing. All participants were healthy and did not present any neurodevelopmental or learning disorders, as reported by their teachers. Participants were Italian citizens from the general population, drawn from an urban environment with a medium socioeconomic status. All the children's parents gave written informed consent prior to testing. The study was approved by the Liguria Regional Ethics Committee (2/2020—DB id 10213) and was conducted in accordance with the Declaration of Helsinki.

**TABLE 1:** Number of participants by age group (*F* = number of females) from recruitment to statistical analyses.

	6yo	7yo	8yo	9yo	10yo
Number of participants that took part at the study	37 (20*F*)	30 (17*F*)	24 (12*F*)	24 (7*F*)	21 (14*F*)
Number of participants excluded a priori	9*F*, 5M	8*F*, 6M	7*F*, 2M	1*F*, 5M	0
Number of participants excluded based on the JND criterium	1M	1*F*	0	1 M, 1*F*	1 M, 1*F*
Final sample for the analyses	22 (11*F*)	15 (8*F*)	15 (5*F*)	16 (5*F*)	19 (13*F*)

### 2.2 Apparatus and Stimuli

The experimental setup included a horizontal rail, a PC, and a stimulation device. The horizontal rail was a 140 cm long home‐built rail on which children could place their arm to support it during movement. The rail allowed a maximum lateral arm displacement of 65 cm.

**FIGURE 1:**
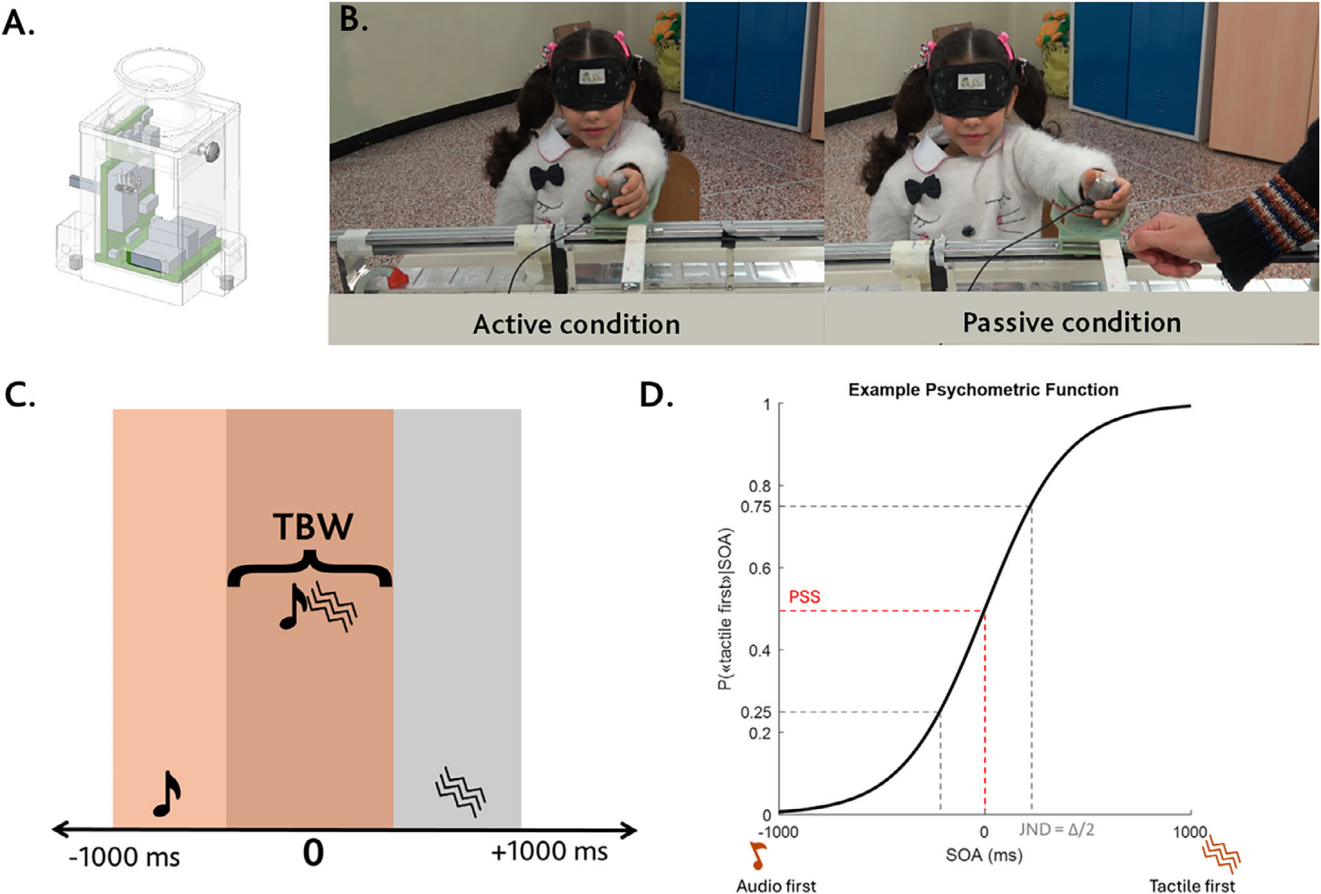
Experimental setup and quantification. (A) shows a module of the Caterpillar device, which provided auditory and vibrotactile stimuli. (B) shows the experimental setup and the experimental conditions (i.e., active and passive). (C) shows the timing of experimental stimuli. Colors represent the time intervals between auditory and tactile stimuli within the temporal binding window (TBW). (D) example of a psychometric function showing how the just noticeable difference (JND) and the point of subjective simultaneity (PSS) were extracted from the cumulative Gaussian function.

The auditory and tactile stimuli were provided using a module of the Caterpillar device (see Figure 1A) that was positioned in the child's hand. The MSI Caterpillar is a compact system developed at the Italian Institute of Technology and is designed to deliver auditory, visual, and tactile stimuli (Gori et al. 2019). In this study, it was used to present both auditory and tactile stimuli from a single, unified source. Although the vibrating motor inherently produces a slight mechanical noise, this is significantly attenuated by sound‐absorbing materials incorporated into the device. The auditory and tactile stimuli had a duration of 10 ms, with the tactile stimulus having a 230 Hz vibration and the auditory stimulus being a 1000 Hz pure tone. The intensity of vibrotactile stimulation ranged between 2 and 3.8 G, depending on how tightly the device was held. Both the auditory and tactile stimuli were generated and temporally controlled by MATLAB 2019 (Math‐Works, Natick, MA, USA) with the Psychtoolbox‐3 package (Brainard 1997; Kleiner et al. 2007; Pelli 1997). All timings were verified with an oscilloscope prior to testing.

### 2.3 The Experimental Paradigm

Participants were blindfolded and performed an audiotactile TOJ task while they moved their arm either actively (i.e., active condition) or while it was passively moved (i.e., passive condition) (see Figure 1B). In the TOJ paradigm, two stimuli (one auditory and one tactile) are presented, separated by a Stimulus Onset Asynchrony (SOA) that varies across trials. Participants are asked to judge which stimulus occurred first and report it verbally to the experimenter. The task was explained to children in a similar way as it would be to adults, maintaining a clear and straightforward instruction style. The only adjustment made concerned terminology: since the terms “sound” and “vibration” were sometimes difficult for children to pronounce, we followed them by the respective onomatopoeic expressions: *beep* to indicate the sound and *brrr* to indicate the vibration.

Negative SOAs represent auditory‐leading trials and positive SOAs represent tactile‐leading ones. The SOA was determined adaptively by a QUEST routine on a trial‐by‐trial basis. QUEST is an adaptive algorithm that uses a Bayesian approach to set the SOA using all the information available from previous trials as well as prior knowledge from the literature (Watson 2017; Watson and Pelli 1983). We used the function QuestCreate of Psychtoolbox‐3 setting the following parameters: tGuess = 0.5, tGuessSd = 0.5, pThreshold = 0.5, beta = 3.5, delta = 0.01, gamma = 0, grain = 0.01, range = 1.

The SOA ranged from 0 ms to 1000 ms (Figure 1C). The task consisted of 120 trials, 60 of which were active trials and 60 passive trials. A secondary experiment was performed on a subgroup of participants to determine whether the observed difference was specific to the comparison between active and passive movement conditions or whether the absence of movement led to improved performance. Specifically, a subgroup of the 6‐year‐old participants (*N* = 13) was administered an additional 30 control trials during which the children were asked to perform the TOJ task without any movement (i.e., static condition). The conditions were administered in a random order for each participant to control for order effects.

### 2.4 Procedure

The child sat in front of a table on which a horizontal rail was placed. The participant's height was adjusted so that their arm formed a 90° angle with the body. Also, the dimensions of arm length, forearm length, distance from the table, and motor span were measured. In the static condition, participants completed the TOJ task, responding to the same question without any arm movement. During these trials, the hand remained still, positioned at the body midline. During the active and passive conditions, the child's left arm moved horizontally with the arm constrained along a straight line by the rail on which the arm was placed. During the active condition, the child controlled the movement of his/her arm, while during the passive condition, an experimenter moved the child's arm. The length of the movement was adapted to the motor span of each participant (range = 20.5–48 cm; mean ± SD = 31.5 ± 6.4 cm). The first stimulus was delivered by the experimenter to coincide with approximately the midpoint of the movement trajectory. In the passive condition, the experimenter manually controlled the child's limb movement while simultaneously triggering the stimulus. In the active condition, the experimenter provided a verbal “go” signal to initiate the child's self‐generated movement, with stimuli delivered at the midpoint. To ensure that the children understood the instructions, they were first familiarized with the stimuli and were then given a brief training session with feedback. Once it was ascertained that each child understood the task, the experiment started. No feedback was provided during the experimental trials and participants had the option to skip trials if they did not perceive the stimuli or became distracted. Skipped trials did not contribute to the QUEST algorithm, and the subsequent trial was presented at the same intensity level as the preceding one, rather than treating the skip as an incorrect response or advancing to a different level. Participants were encouraged to take breaks whenever needed. Without breaks, each condition took approximately 5 min to complete.

### 2.5 Quantification and Statistical Analysis

Statistical analyses were carried out in the R environment (R version 4.1.2, 2021‐11‐01) using RStudio (version 2021.09.1, Build 372) (R Core Team 2024).

#### 2.5.1 Calculation of the Psychometric Curve

Based on participants’ judgments, the proportion of “which stimulus came first” responses was plotted as a function of SOA for each participant and condition. A cumulative Gaussian function was fitted to the data to estimate the point of subjective simultaneity (PSS) and just noticeable difference (JND). The PSS represents the perceptual bias of a participant's perception of auditory‐tactile synchrony, while the JND represents the smallest temporal difference between auditory and tactile signals that an individual could detect and is an index of precision. The JND is defined as half the difference between the SOAs corresponding to response probabilities of 0.75 and 0.25 (Figure 1D). *R* ^2^ has been calculated as a measure of goodness of fit.

#### 2.5.2 Outlier Removal and Trials

Outliers were identified and removed based on a JND greater than two standard deviations above the group mean for each condition (passive, active). The final sample was composed as follows:

Behavioral data were cleaned by excluding trials in which participants either failed to perceive the stimuli or became distracted. As a result, participants completed on average 52.5 ± 12.8 trials in thective condition (range = 14–60), and 52.8 ± 12.1 trials in the passive condition (range = 21–60).

#### 2.5.3 Statistical Analysis

Statistical analyses were performed to explore the developmental trend of the audiotactile temporal precision (JND) and bias (PSS) and the impact of passive and active movement on it. First, for both JND and PSS, the normality of the data distribution was assessed separately for each condition (active and passive) with Shapiro‐Wilk normality tests. Since the data were not all normally distributed (for JND active: *W* = 0.95, *p* = 0.003; JND passive: *W* = 0.92, *p* < 0.001; PSE active: *W* = 0.97, *p* = 0.08; PSE passive: *W* = 0.91, *p* < 0.001), we performed permutation‐based tests for factorial and repeated‐measures ANOVA using the *aovperm* function from the *permuco* package (Frossard and Renaud 2021). Unlike standard parametric *t*‐tests, which rely on assumptions about the normal distribution of the data, permutation *t*‐tests are non‐parametric and do not require such assumptions. Instead, they estimate the null distribution by repeatedly reshuffling the group labels and recalculating the test statistic, providing a robust inference method particularly suitable for small samples or non‐normally distributed data. The dependent variables (JND and PSS) were tested as a function of condition and age group. The model formula used was:

JND∼Condition∗AgeGroup+Error(Subject/Condition)

PSS∼Condition∗AgeGroup+Error(Subject/Condition)

Follow‐up analyses included permutation *t*‐tests based on 9999 permutations performed with the *perm.t.test* function from the *MKinfer* package (Kohl 2024). Moreover, to assess whether the PSS significantly differed from perfect synchrony (0 ms), we conducted one‐sample permutation *t*‐tests separately for each age group. All results were corrected for multiple comparisons using the Bonferroni method. Since an effect of age was observed, a linear mixed‐effects model was fitted to explore changes in JND and PSS as a function of continuous age and condition, using the *lmer* function from the *lme4* package (Bates et al. 2015):

JND∼Age∗Condition+(1|Subject)

PSS∼Age∗Condition+(1|Subject)

Because the residuals were not uniformly distributed, the model results were evaluated with a permutation ANOVA using the *permanova.lmer* function from the *predictmeans* package (Luo et al. 2024). Additionally, data for each condition were fitted and tested using linear models with permutation tests, with the *lmp* function of the package *lmPerm* (Wheeler and Torchiano 2025).

Similar statistical analyses were performed for the subgroup of 6‐year‐old participants who also completed the static condition data were analyzed separately using nonparametric methods due to violations of normality assumptions (*W* = 0.86, *p* = 0.04). A permutation‐based ANOVA was conducted (*aovperm* function) to assess the effect of age group on JND and PSS, and follow‐up comparisons between age groups were performed using permutation *t*‐tests (*perm.t.test*). Results were corrected for multiple comparisons using the Bonferroni method.

## 3 Results

### 3.1 Development of Audiotactile TOJ Precision During Active and Passive Movements

For each age group and condition (active, passive), we calculated *R* ^2^ as a measure of goodness of fit of the psychometric functions (see Table 2).

**TABLE 2:** *R* ^2^ (mean ± standard deviation) for the psychometric functions of each age group and condition.

	6yo	7yo	8yo	9yo	10yo
Active	0.23 ± 0.15	0.48 ± 0.2	0.4 ± 0.23	0.49 ± 0.2	0.38 ± 0.22
Passive	0.21 ± 0.17	0.49 ± 0.17	0.38 ± 0.18	0.46 ± 0.16	0.37 ± 0.21

**FIGURE 2:**
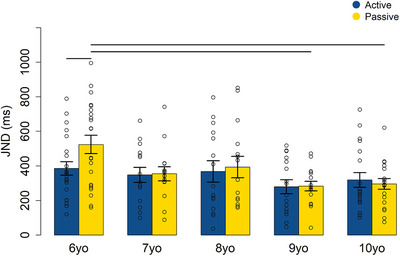
Mean JNDs ± standard error of the mean (SEM) for each age group in the active (blue) and passive (yellow) conditions. Dots represent individual data points; lines indicate significant differences.

The permutation‐based ANOVA on JND revealed a significant main effect of age group (*F*(183) = 9.81, *p* = 0.001, *η* ^2^ = 0.08) and condition (*F*(183) = 9.07, *p* = 0.004, *η* ^2^ = 0.02), as well as a significant age group *x* condition interaction (*F*(183) = 7.36, *p* = 0.007, *η* ^2^ = 0.02). Follow‐up permutation tests based on 9999 permutations showed that a significant difference between the active and passive conditions was found only in the 6‐year‐old group (mean of the differences (SE) = −138.13(36.87), *p* = 0.02, 95% CI = [−226.4, −50.24]; see Table S1). Specifically, in the 6‐year‐old children, the JND in the passive condition was significantly higher than in the active condition (see Figure 2). In line with this, precision in the passive task improved with age, with significantly better performance in 9‐ (mean of the differences (SE) = −241.2(76.62), *p* = 0.004, 95% CI = [−362.27, −118.6] and 10‐ (mean of the differences (SE) = −229.61(72.65), *p* = 0.008, 95% CI = [−353.22, −103.74] year‐olds compared to 6‐year‐olds (see Table S2).

**FIGURE 3:**
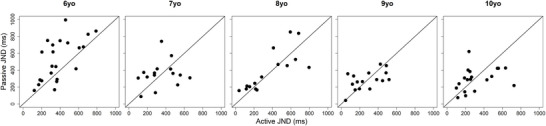
Passive individual JNDs plotted against active individual JNDs for each age group.

In Figure 3, individual JNDs in the passive condition are plotted against those in the active condition for each age group. Older participants show similar performance across the two conditions, with most data points clustering around the equality line (i.e., the diagonal). In contrast, 6‐year‐old participants exhibit discrepancies between conditions: nearly all data points lie above the equality line, suggesting higher precision in the active condition compared to the passive condition.

**FIGURE 4:**
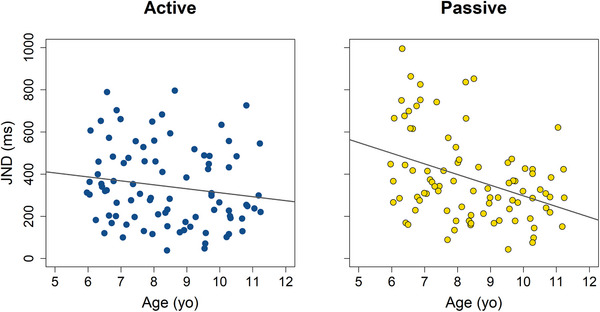
Individual JNDs plotted as a function of age for the active (left) and passive (right) conditions. No significant age‐related change in JND was observed in the active condition (*β* = −18.76, *R* ^2^ = 0.02, *p* = 0.15). In contrast, JND decreased significantly with age in passive condition (*β* = −50.4, *R* ^2^ = 0.14, *p* = 0.0003).

To further examine the relationship between age and movement condition, age was treated as a continuous variable. A permutation‐based ANOVA on the linear mixed‐effects model (JND ∼ Age * Condition + (1|Subject)) revealed a significant interaction between age and condition (*F*(186.34) = 5.58, *p* = 0.03), as well as significant main effects of age (*F*(1, 88.93) = 12.4, *p* = 0.02) and condition (*F*(186.34) = 2.97, *p* = 0.001). To explore the age *x* condition interaction, separate linear models were fitted for the active and passive conditions using age as a continuous predictor (see Figure 4). Permutation‐based linear regressions revealed that in the passive condition, JND decreased significantly with age (*β* = –50.4, *R* ^2^ = 0.14, *p* = 0.0003; 5000 permutations), suggesting improved temporal precision. In contrast, no significant age‐related change in JND was observed in the active condition (*β* = −18.76, *R* ^2^ = 0.02, *p* = 0.15). These results suggest that age‐related improvements in TOJ precision are more pronounced during passive movement.

### 3.2 Development of Audiotactile TOJ Bias During Active and Passive Movements

**FIGURE 5:**
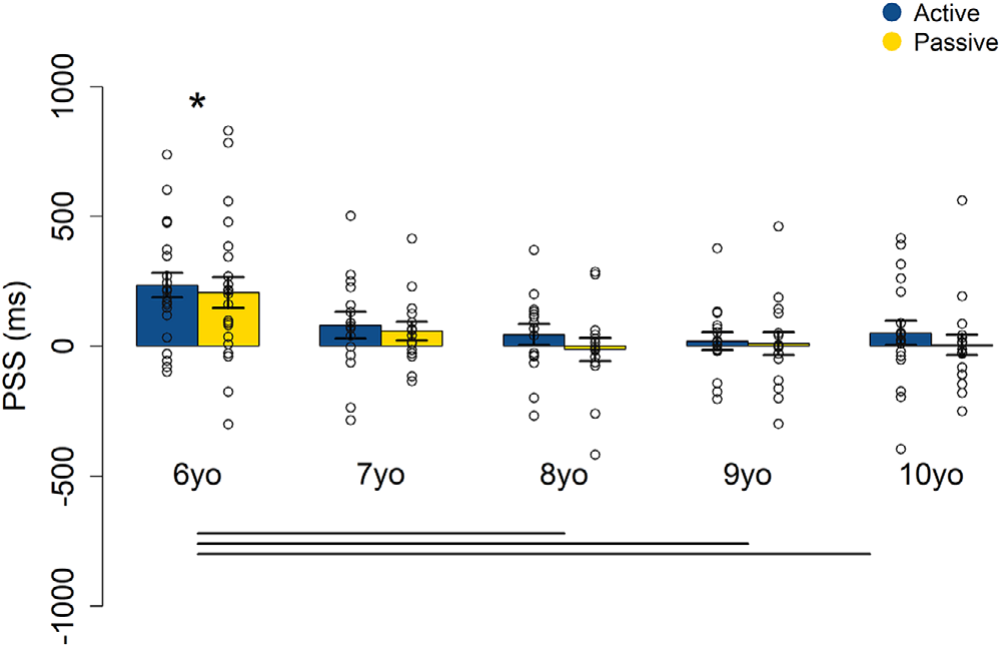
Mean PSSs ± SEM for each age group. 0 ms indicates synchrony between the auditory and tactile stimuli; positive values indicate that the tactile stimulus occurred first, while negative values indicate that the auditory stimulus occurred first. Dots represent individual data points; lines indicate significant differences; *indicates that the mean PSS is significantly different from 0.

The permutation‐based ANOVA on PSS revealed a significant main effect of age group (*F*(183) = 14.15, *p* < 0.001, *η* ^2^ = 0.1), a non‐significant main effect of condition (*F*(183) = 0.01, *p* = 0.9, *η* ^2^ < 0.001), and no evidence for a significant age group *x* condition interaction (*F*(183) = 0.04, *p* = 0.8, *η* ^2^ < 0.001). Follow‐up permutation tests to compare groups demonstrated a significant difference in PSS between the 6‐year‐old group and the 8‐year‐olds (mean of the differences (SE) = ‐206.06 (72.46), CI = [−327.48, −82.77]; *p* = 0.01), 9‐year‐olds (mean of the differences (SE) = −206.07 (71.01), CI = [−327.06, −86]; *p* = 0.01), and 10‐year‐olds (mean of the differences (SE) = −192.86 (67.88), CI = [−314.91, −71.8]; *p* = 0.02), respectively. Table S3 and Figure 5 show mean PSE and standard error of the mean (SEM) for each age group. Only in the 6‐year‐old age group did the permutation test reveal that the PSS significantly deviated from 0 ms, which represents objective synchrony between the auditory and tactile stimuli (Mean of the differences (SE) = 221.32 (40.06, CI = [123.33, 318.85]; *p* = 0.001). The shift in PSS from positive values to approximately 0 ms with age suggests that temporal perception becomes more calibrated toward synchrony across both movement conditions as age increases, although a tactile‐leading shift is still maintained. This result is further confirmed by the permutation‐based analyses considering age as a continuous variable. Specifically, the permutation‐based ANOVA on the linear mixed model revealed a significant main effect of age only (*F*(1, 86.69) = 16.7, *p* = 0.001), and the subsequent permutation‐based linear regression showed a decrease in PSS with increasing age (*β* = −41.95, *R* ^2^ = 0.1, *p* < 0.01).

### 3.3 Control Comparisons With the Static Condition

**FIGURE 6:**
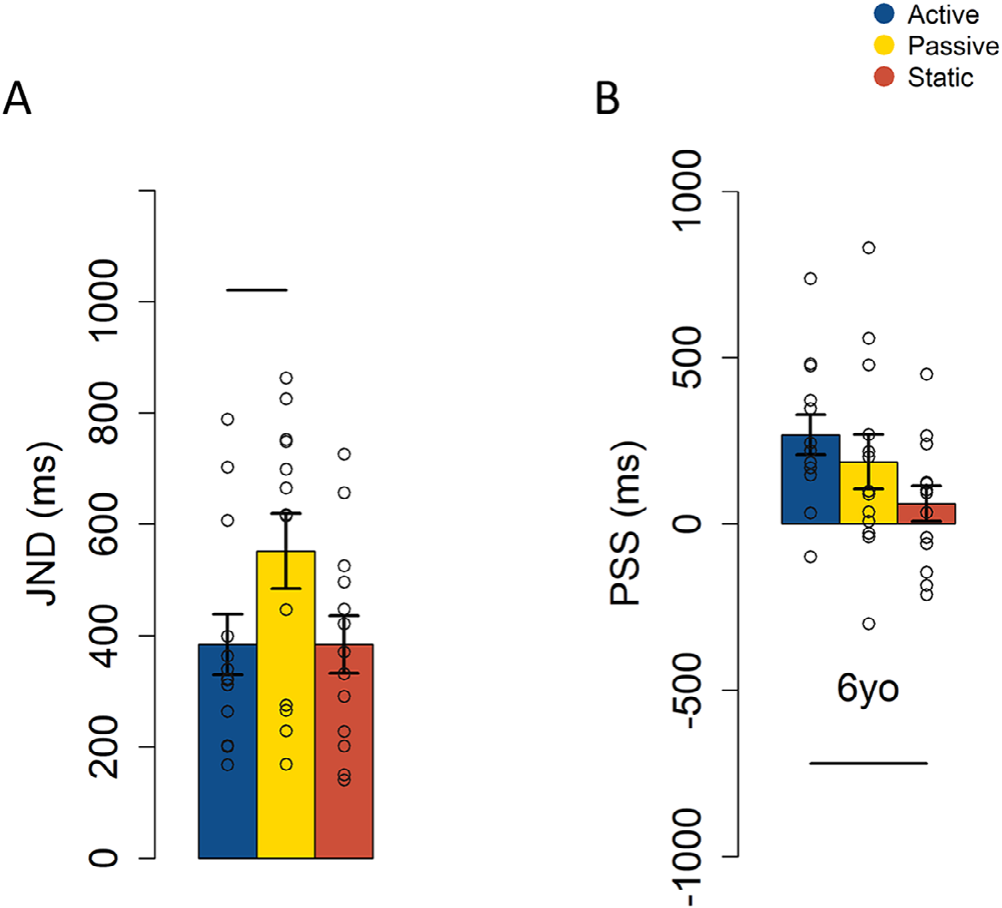
A. Mean JNDs ± SEM for the 6‐year‐old subgroup that also performed the static condition. Dots represent individual data points; lines indicate significant differences. Blue represents the active condition; yellow represents the passive condition; red represents the static condition. B. Mean PSSs ± SEM for the 6‐year‐old subgroup that also performed the static condition. 0 ms indicates synchrony between the auditory and tactile stimuli; positive values indicate that the tactile stimulus occurred first, while negative values indicate that the auditory stimulus occurred first. Dots represent individual data points; lines indicate significant differences. Blue represents the active condition; yellow represents the passive condition; red represents the static condition.

A subgroup of thirteen 6‐year‐old children also completed the static condition to determine whether the observed difference was specific to the comparison between active and passive movement conditions or whether the absence of movement led to improved performance. The permutation‐based ANOVA on JND revealed a significant effect of condition (*F*(224) = 3.72, *p* = 0.036, *η^2^* = 0.1). Follow‐up analyses confirmed the main result: better precision in the active condition compared to the passive condition persists even within this smaller sample (mean of the differences (SE) = −167.64 (53.48), CI = [−270.76, −64.03], *p* = 0.01; see Figure 6A). The static condition did not differ significantly from the active condition (mean of the differences (SE) = 0.4 (71.99), CI = [‐139.47, 138.66], *p* = 0.99), suggesting that even though the mechanism supporting enhanced temporal precision during active movement begins to develop at this age, it remains immature, likely due to high motor noise. Indeed, performance differences are evident relative to the passive condition but not the static condition. This may indicate that the predictive mechanism is sufficiently developed to compensate for the motor noise associated with active movement, maintaining performance levels comparable to the no movement condition. However, it appears not yet mature enough to fully overcome this noise and provide the additional performance benefit. Moreover, although Figure 6A visually suggests poorer precision in the passive compared to the static condition, the permutation‐based *t*‐test corrected for multiple comparisons (*n* = 3) did not reveal a statistically significant difference (Mean of the differences (SE) = ‐168.04(76.65), CI = [−317.11, −18.89], *p* = 0.17). This lack of statistical significance is likely due to the small sample size in this control experiment (*N* = 13). As for the PSS (Figure 6B), the permutation‐based ANOVA demonstrated a significant effect of the condition (*F*(224) = 6.58, *p* = 0.005). Subsequent permutation‐based *t*‐tests show a significant difference between the active and the static conditions (mean of the differences (SE) = 207.15(46.51), CI = [−115.27, 298.7], *p* = 0.004), suggesting that the shift from 0 ms is reduced for the static condition. The difference between passive and static conditions was not significant after Bonferroni correction for multiple comparisons (Mean of the differences (SE) = −125.37 (51.07), CI = (−220.76, −29.09], *p* = 0.08).

## 4 Discussion

This study explored the developmental trajectory of the impact of active and passive motion on the audiotactile temporal binding window (TBW). Active movement increased the precision of temporal order judgments in relation to passive movement. Crucially, the active‐passive difference was evident at 6 years of age but not at the older ages. The primary effect observed at the older ages was increasing temporal precision with age in the passive condition (i.e., the JND decreased as a function of age). Overall, temporal bias (i.e., PSS) shows a similar developmental trajectory in both active and passive conditions, with a reduction of the shift toward the tactile modality (i.e., closer to objective simultaneity) with age.

### 4.1 Development of Temporal Precision During Active and Passive Movement

The first result shows that active movement reduces the temporal precision compared to passive movement in 6‐year‐old children, which is similar to prior observations in adults (Kitagawa et al. 2016; Nishi et al. 2014; Tanaka et al. 2021). However, this difference was absent in older children, suggesting a specific importance of the 6‐year‐old age. As detailed in Section 4.3, this age appears to constitute a critical developmental milestone that modulates temporal perception and sensorimotor integration.

These findings suggest that the brain begins to utilize efference copy signals early in development to facilitate motor prediction, thereby yielding measurable behavioral benefits. However, results of a control experiment suggest similar performance between active and static conditions in the 6‐year‐olds, raising the hypothesis that PSF is sufficiently developed to compensate for motor noise, but it is not yet mature enough to fully overcome it and provide an additional benefit.

Moreover, even though active movement facilitates JNDs in young children, suggesting early PSF development, results across all examined age ranges indicate that the primary mechanism undergoing maturation during this period is not the sensorimotor interaction producing the predicted sensory feedback (PSF). Instead, maturation more likely occurs in the ability to integrate multisensory signals with perceived movement. Consistent with this conclusion is the fact that the processing of external sensory feedback throughout childhood produces imprecise input for sensory integration and that this ability only reaches adult‐like levels during adolescence (Gori et al. 2012). Therefore, it is possible that the network involved in the integration of noisy afferent sensory signals with movement requires sufficient maturation before the internal feedforward dynamics model can generate a PSF that optimizes multisensory integration processes. This is further supported by the fact that the maturation of sensorimotor integration signals during voluntary movement for unisensory stimuli is completed in late childhood, with a lag relative to the development of intersensory integration (Gori et al. 2012).

### 4.2 The Influence of Motion on the Point of Subjective Simultaneity Changes With Development

The bias results also support the idea that multisensory mechanisms mature earlier than those relying on sensory feedback. Indeed, although at age six the brain begins to employ motor prediction as suggested by the precision results, this mechanism is not yet sufficiently effective to correct temporal biases, as indicated by a tactile leading shift of the PSS. Thus, in young children, the tactile cue needs to be presented earlier for it to be perceived as simultaneous with the auditory cue, as previously reported (Ampollini et al. 2024; Stanley et al. 2019), reflecting the relative processing times of the signals in each modality (García‐Pérez and Alcalá‐Quintana 2012). With increasing age, audiotactile temporal perception moves closer to objective simultaneity in both movement conditions, even though a slight tactile‐leading shift is maintained. As previously reported (Ampollini et al. 2024; Stanley et al. 2019), this suggests that the PSS has not yet reached adult‐like levels at 6 years of age and only begins to stabilize starting around 7 years of age.

The similarity of the developmental changes that occur during active and passive movements is consistent with the notion that the emergence of mature systems occurs predominantly within systems that are common between the two conditions (Kitagawa et al. 2016). Specifically, the observed effects appear to be driven more by the development of processes that integrate sensory signals (e.g., auditory and tactile) with perceived movement (i.e., proprioceptive input), rather than by the development of the sensorimotor integration system with the generation of the PSF that is specific only to the active condition. This interpretation is reinforced by evidence from adults in whom active movement leads to a reversal in the temporal order of audiotactile perception (Hao et al. 2015; Nishi et al. 2014). That is, in adults, the auditory stimulus must precede the tactile stimulus for the two to be perceived as simultaneous during active movement, with the opposite pattern for passive movements.

### 4.3 The Development of Time Perception and the Age of Six

One question that arises from the results is, if these mechanisms are not yet fully mature, why do we still observe a movement‐related benefit at 6 years of age? The answer to this question most likely lies in the fact that this particular age represents a critical milestone in child development. It marks the transition from the preschool to the school period, and it is characterized by significant changes in the development of time perception (Buzi et al. 2024; Droit‐Volet 2013).

During the preschool years, brain maturation involves myelination of cortico‐cortical tracts and a decrease in cortical thickness, particularly in prefrontal and parietal areas. This is accompanied by transient grey matter volume increases in prefrontal and cerebellar regions (Brown and Jernigan 2012). Functionally, while the resting state network remains immature, early connectivity between dorsal medial frontal and posterior regions emerges. Moreover, there is an increase in structural network efficiency. All these neural changes may underlie the development of temporal perception across sensory modalities (Buzi et al. 2024). Nonetheless, at this developmental stage, time is still linked to experience and events but is not yet an abstract concept enabling children to voluntarily focus their attention on the flow of time. Thus, during the preschool years, time judgment is more implicit, and it is only later that children acquire explicit knowledge about time and temporal relations (Droit‐Volet 2013). Considering that implicit time perception arises from sensorimotor information, the temporal perception that is acquired through motor rhythm appears to be essential during this period of development. In essence, it further enhances motor skills by supporting movement coordination and manipulation of moving objects (Coull and Nobre 2008; Monier and Droit‐Volet, 2019). Moreover, language development during this period also helps children to link objects and events. The acquisition of deictic terms (yesterday, tomorrow) and durative terms (minute, hour) around age 3–4 begins to allow the children to “decouple” time from the immediate event (Weist 1989). Before this, time is inextricably linked to the present action (e.g., “time to eat”). Moreover, words like “before” and “after”, acquired around age 5, provide the logical structure necessary to begin contextualizing experiences explicitly in terms of time (Hudson and Mayhew 2011). During the school years, cortical grey matter increases until around age 10 and then thins with age (especially in parieto‐temporal regions) due to synaptic pruning, while the frontal grey matter continues to increase in volume (Tau and Peterson 2010). This maturation is also supported by white matter expansion and increased myelination, improving connectivity and functional network communication (Buzi et al. 2024). These maturational dynamics, along with the rich exposure to diverse stimuli and structured learning during the first school years, foster the development of fluid cognitive skills and expand the vocabulary (Nagy et al. 2004).

Temporal sensitivity improvement with age has been associated with the development of short‐term memory span and attention/executive functions (Zélanti and Droit‐Volet 2011), which are themselves mutually influenced by language acquisition (Schweppe et al. 2022; Shokrkon and Nicoladis 2022). It can be hypothesized that if a child possesses precise labels to define time intervals, they are better at maintaining temporal information in short‐term memory during a task, thereby improving performance. Furthermore, language expansion fosters a more accurate temporal concept. Indeed, associating time‐related words with the perception of duration does not come naturally to children; early intuitive meanings of time terms are instead based on relative orderings, which children infer from their usage in speech (Tillman and Barner 2015). Additionally, poorer temporal sensitivity and working memory have been observed in children with reading and oral language difficulties (Beattie and Manis 2012; Larson et al. 2023). Collectively, the development of these skills also facilitates the acquisition of explicit temporal judgments and time‐related concepts (Droit‐Volet 2013).

### 4.4 General Developmental Mechanisms

**FIGURE 7:**
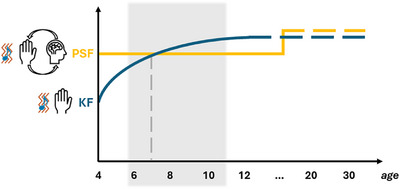
Schematic representation of the two mechanisms as a function of age. The sensorimotor integration involving the predicted sensory feedback (PSF) is represented in yellow as specifically involved in the active movement condition. The multisensory integration of auditory and tactile stimuli aligned with kinesthetic feedback (KF) is represented in blue as it is observable in the passive condition. The grey area represents the ages investigated in the current study.

Beyond the development of time perception, the maturation of the audiotactile TBW during movement seems to involve two main mechanisms: the sensorimotor integration involving the PSF, as well as the development of multisensory integration of auditory and tactile stimuli aligned with kinesthetic feedback (KF) provided by proprioceptive signals from the movement itself (Kitagawa et al. 2016). Based on our results, we can speculate that these two mechanisms follow different developmental trajectories and that they emerge at different points in development (Figure 7). Sensorimotor integration seems to have an early tuning phase (during the preschool period) that precedes aspects of multisensory maturation. It allows active movement to provide a benefit over passive movement at 6 years of age and sustains implicit time judgments. Importantly, however, this process seems to slow down during the school years only to resume further tuning later in development. Once this process is completed, the adult‐like benefit of movement for audiotactile temporal perception emerges. In contrast to the development of sensorimotor integration involving the PSF, the development of multisensory integration aligned with KF seems to follow a faster developmental trajectory within the school‐age period. The shift to the acquisition of explicit time judgment also reduces the benefit of active movement. It is plausible that the proper maturation and alignment of sensory systems in a growing body is necessary for sensorimotor integration to enter a later tuning phase and that, once this happens, the benefits of active movement can reemerge. Although sensorimotor and multisensory integration are hypothesized as the primary factors driving the observed pattern based on our experimental design, we cannot exclude that other maturational mechanisms may also contribute to task performance. For instance, attentional control and susceptibility to movement‐related distraction cannot be fully disentangled in pediatric populations, where top‐down regulation matures gradually. Similarly, task engagement and working memory load may covary with age, affecting multisensory and sensorimotor performance. These factors align with established developmental trajectories in executive function. Future studies should incorporate targeted manipulations (for e.g., introducing unrelated distractions) to decompose attentional confounds from genuine predictive sensory feedback (PSF). Larger samples across finer age bins would also enhance statistical power to detect interaction effects. While our design provides preliminary evidence for PSF maturation, these complementary mechanisms represent testable hypotheses rather than nullified alternatives, guiding a more comprehensive model of sensorimotor development. In conclusion, our results suggest the presence of a sequence of tuning or plastic phases followed by periods of relative stability. It is possible that this sequence reflects the need to align the maturation of sensory systems with the uneven growth of the body and the concurrent development of the motor system. Additional studies are needed to validate this hypothesis and to fully elucidate these developmental trajectories. The comprehension of these developmental trajectories will provide greater insights into the mechanisms that go awry in neurodivergent and psychiatric populations (Ampollini et al. 2024; Wallace and Stevenson 2014) and how different abilities, such as sport activities or languages can impact them. Once such trajectories and mechanisms are elucidated, it will be possible to design optimal interventions to improve the quality of life in these populations.

### 4.5 Limitations of the Study

Although the current findings offer valuable insights into the developmental interplay between sensorimotor and audiotactile multisensory integration, some limitations remain and highlight the need for further investigation. (i) We did not include an adult comparison group. This makes our hypothesis of a U‐shaped developmental trajectory for active movement benefits speculative. Nonetheless, it should be noted that different studies with adults have reported active movement benefits in the audiotactile TOJ task (Kitagawa et al. 2016; Nishi et al. 2014; Tanaka et al. 2021). (ii) We did not include a static condition in all age groups, and the sample in this condition at 6‐years of age is smaller. However, the focus of this study was on the comparison between active and passive movement, and unfortunately, children struggled to complete all conditions. Moreover, the study uses a within‐subjects design, which substantially increases statistical power by minimizing inter‐individual variability, along with the use of a permutation‐based ANOVA, which provides reliable p‐values even with small samples. The medium effect size for the condition factor in the control experiment (*η* ^2^ = 0.10) further confirms the significant result is not merely noise from the small sample. (iii) Our experimental setup did not include a precisely controlled haptic system. Even though this contributed to increased variability in inter‐trial timing and stimulus timing during movement, it also may have reduced the possibility of trial‐by‐trial prediction and may have allowed for a clearer dissociation between active and passive movement mechanisms. (iv) We did not control movement speed, which is known to influence the TBW (Droit‐Volet 2013; Vannucci et al. 2025). Although future research should incorporate this variable, no study to date has explored the role of movement speed in modulating active versus passive conditions within the audiotactile TOJ task. (v) We did not include unrelated distraction conditions to directly assess the role of diverted attention. Future studies should incorporate such manipulations to better determine whether the observed effect reflects attentional distraction rather than the motor prediction mechanism alone.

## 5 Conclusion

The current study shows the effect of active and passive movement on the development of audiotactile temporal perception and suggests a synergistic, though temporally dissociated, involvement of the sensorimotor process involving the PSF and multisensory integration process aligned with KF. The early benefit of active movement diminishes as children shift from implicit to explicit time judgments, reemerging later when predictive timing abilities fully mature. Overall, the study highlights a pattern of developmental tuning phases and emphasizes the importance of aligning sensory and motor system maturation. Future studies are needed to confirm our results and the involvement of other movement variables.

## Funding

This research was conducted as part of the Multisensory Environments to study Longitudinal Development (MELD) Consortium. The work of MELD is supported by a generous unrestricted gift from Reality Labs Research, a division of Meta.

## Ethics Statement

The study was approved by the Liguria Regional Ethics Committee (2/2020 – DB id 10213) and was conducted in accordance with the Declaration of Helsinki.

## Conflicts of Interest

The authors declare no competing interests.

## AI disclosure Statement

ChatGPT‐5.4 was used in the introduction and discussion to edit and rephrase some sections for clarity and conciseness, suggest alternative word choices, identify redundant content, or improve transitions between sections. AI‐generated editing was reviewed, validated, and cross‐checked for accuracy. The influence on the conclusion was quite insignificant, as AI was only used to improve the clarity but without altering authors' considerations and conclusions.

## Supporting information




**Supporting File 1**: desc70191‐sup‐0001‐SuppMat.docx

## Data Availability

The data will be uploaded to Zenodo upon acceptance of the manuscript.
