# Supplementary material for: The Role of Movement on the Development of the Audiotactile Temporal Binding Window
Source: Dev Sci. 2026 Apr 13;29:e70191. doi: 10.1111/desc.70191 (PMC13075528; doi:10.1111/desc.70191)
Supplement: Supplementary file 1 — Supporting File 1: desc70191‐sup‐0001‐SuppMat.docx [file DESC-29-e70191-s001.docx]

# Supplemental Materials

# Tables

| **Active vs. Passive** | **Mean of the differences**  **(Standard Error - SE)** | **95% Confidence Interval (CI)** | **p-value corrected** |
| --- | --- | --- | --- |
| 6yo | -138.13(36.87) | [-226.4, -50.24] | 0.02 * |
| 7yo | -6.22(49.19) | [-120.24, 107.3] | 1 |
| 8yo | -24.94(36.49) | [-111.49, 61.36] | 1 |
| 9yo | -3.46(32.74) | [-75.59, 68.59] | 1 |
| 10yo | 23.31(42.26) | [-67.91, 114.55] | 1 |

**Table S1. Results of the permutation-based t-tests comparing Active and Passive conditions between each age Group.**

| **ACTIVE CONDITION** | | | |
| --- | --- | --- | --- |
| **Contrasts** | **Mean of the differences**  **(Standard Error - SE)** | **95% Confidence Interval (CI)** | **p-value corrected** |
| 6yo vs. 7yo | 38.03(58.67) | [-81.28, 156.42] | 1 |
| 6yo vs. 8yo | -17.79(68.59) | [-168.6, 133.6] | 1 |
| 6yo vs. 9yo | -105.32(58.86) | [-219.11, 7.84] | 0.6 |
| 6yo vs. 10yo | -66.72(57.87) | [-183.7, 50.01] | 1 |
| 7yo vs. 8yo | 18.82(74.6) | [-136.08, 176.22] | 1 |
| 7yo vs. 9yo | -67.59(59.34) | [-189.1, 52.97] | 1 |
| 7yo vs. 10yo | -28.41(60.84) | [-153.41, 94.85] | 1 |
| 8yo vs. 9yo | -87.37(73.52) | [-240.7, 64.44] | 1 |
| 8yo vs. 10yo | 50.35(72.47) | [-204.29, 105.6] | 1 |
| 9yo vs. 10yo | 38.96(58.79) | [-80.31, 157.89] | 1 |
| **PASSIVE CONDITION** | | | |
| **Contrasts** | **Mean of the differences**  **(Standard Error - SE)** | **95% Confidence Interval (CI)** | **p-value corrected** |
| 6yo vs. 7yo | 169.28(76.8) | [33.93, 304.91] | 0.18 |
| 6yo vs. 8yo | -130.77(83.63) | [-296.78, 35.27] | 1 |
| 6yo vs. 9yo | -241.2(76.62) | [-362.27, -118.63] | 0.004* |
| 6yo vs. 10yo | -229.61(72.65) | [-353.22, -103.74] | 0.008 * |
| 7yo vs. 8yo | 38.3(73.02) | [-113.98, 191.314] | 1 |
| 7yo vs. 9yo | -70.89(49.5) | [-172.25, 30.18] | 1 |
| 7yo vs. 10yo | -58.89(50.44) | [-163.77, 45.65] | 1 |
| 8yo vs. 9yo | -110.3(68.16) | [-251.25, 31.85] | 1 |
| 8yo vs. 10yo | -97.84(66.09) | [-241.61, 46.15] | 1 |
| 9yo vs. 10yo | 11.79(41.65) | [-72.5, 96.44] | 1 |

**Table S2. Results of the permutation-based t-tests comparing Age Groups within the Active (top) and Passive Conditions (bottom).**

| **Age Group** | **Mean PSE ± SEM** |
| --- | --- |
| 6yo | 221.09 ± 37.55 |
| 7yo | 69.53 ± 30.64 |
| 8yo | 15.97 ± 30.01 |
| 9yo | 14.56 ± 27.36 |
| 10yo | 27.74 ± 30.1 |

**Table S3. Mean PSE ± SEM for each age group.**
